# Silencing an E3 Ubiquitin Ligase Gene *OsJMJ715* Enhances the Resistance of Rice to a Piercing-Sucking Herbivore by Activating ABA and JA Signaling Pathways

**DOI:** 10.3390/ijms222313020

**Published:** 2021-12-01

**Authors:** Yuebai Zhang, Mengting Chen, Shuxing Zhou, Yonggen Lou, Jing Lu

**Affiliations:** 1 State Key Laboratory of Rice Biology & Ministry of Agriculture Key Lab of Agricultural Entomology, Institute of Insect Sciences, Zhejiang University, Hangzhou 310058, China; 11816019@zju.edu.cn (Y.Z.); mtchen@zju.edu.cn (M.C.); 3090100232@zju.edu.cn (S.Z.); 2 Hainan Institute, Zhejiang University, Sanya 572025, China

**Keywords:** rice, OsJMJ715, *Nilaparvata lugens*, herbivore-induced defenses

## Abstract

The RING-type E3 ubiquitin ligases play an important role in plant growth, development, and defense responses to abiotic stresses and pathogens. However, their roles in the resistance of plants to herbivorous insects remain largely unknown. In this study, we isolated the rice gene *OsJMJ715*, which encodes a RING-domain containing protein, and investigated its role in rice resistance to brown planthopper (BPH, *Nilaparvata lugens*). OsJMJ715 is a nucleus-localized E3 ligase whose mRNA levels were upregulated by the infestation of gravid BPH females, mechanical wounding, and treatment with JA or ABA. Silencing *OsJMJ715* enhanced BPH-elicited levels of ABA, JA, and JA-Ile as well as the amount of callose deposition in plants, which in turn increased the resistance of rice to BPH by reducing the feeding of BPH and the hatching rate of BPH eggs. These findings suggest that OsJMJ715 negative regulates the BPH-induced biosynthesis of ABA, JA, and JA-Ile and that BPH benefits by enhancing the expression of OsJMJ715.

## 1. Introduction

To defend themselves from herbivorous insects, plants have developed sophisticated defensive mechanisms known as constitutive and inducible defenses [1,2]. The inducible defense starts with the perception of herbivore-associated molecular patterns and/or damage-associated molecular patterns, followed by the activation of an array of early signaling events, such as the increase in levels of cytosolic calcium ion, burst of reactive oxygen species (ROS), and activation of mitogen-activated protein kinase (MAPK) cascades. These early signaling events elicit defense-related signaling pathways mediated by phytohormones, which in turn lead to a global reconfiguration of transcriptome, proteome, and metabolome, thereby enhancing direct and/or indirect defenses of plants to herbivores [2]. These phytohormones mainly include jasmonic acid (JA), salicylic acid (SA), and ethylene (ET). Recently, other phytohormones, such as abscisic acid (ABA), gibberellic acids, cytokinins, and auxins, have also been reported to modulate herbivore resistance through synergistically or antagonistically interacting with JA signaling [3]. For instance, Vos et al. [4] found that in *Arabidopsis thaliana*, the activation of primed JA-regulated defenses in response to secondary herbivore attack is dependent on ABA signaling. In contrast, ABA has been shown to negatively regulate resistance to *Hirschmanniella oryzae* by inhibiting JA biosynthesis in rice (*Oryza sativa*) roots [5].

Ubiquitin (Ub) is a polypeptide consisting of 76 amino acids, and its mediated proteolysis extensively exists in eukaryotes. The Ub-targeted proteins are first modified by a cascade of reactions catalyzed mainly by three enzymes involving Ub-activating enzymes (E1), Ub-conjugating enzymes (E2), and Ub ligases (E3); then, the mono- or poly-ubiquitylated proteins are degraded by the 26S proteasome system [6,7,8]. Among these enzymes, E3 ligases are responsible for determining substrate specificity [8]. In *Arabidopsis* and rice, more than putative 1500 and 1300 E3 ligase-encoding genes were predicted, respectively; these E3 ligases can be classified mainly into four subfamilies on the basis of their characteristic domains and catalytic mechanisms: HECT (homologous to E6-associated protein C-terminus), RING (really interesting new genes), U-Box, and CRLs (cullin-RING ligases) [8,9,10]. Unlike other subfamilies of E3 ligases, CRLs incorporate with other proteins to form a complex that binds to substrates [9].

Ub-mediated proteolysis has been reported to play an important role in the defense response of plants to biotic and abiotic stresses by regulating the biosynthesis and signaling of defense-related phytohormones or other defense-related components [11,12]. In *Arabidopsis*, for instance, the CRL-type E3 ligase CRL1 is involved in the signaling of JA by forming the SKP1–CRL1–F-box (SCF) complex assembled with the F-box protein COI1 (SCF^COI1^). This process causes the degradation of JAZ repressors and activates the transcription of JA-responsive genes [13]. Moreover, the U-Box-type E3 ligases PUB22/PUB23/PUB24 negatively regulate PAMP-triggered immunity by modulating the production of ROS and cell death [14], and PUB19 negatively regulates ABA and drought responses [15]. In addition, RING-type E3 ligases have also been reported to regulate plant resistance to diseases and abiotic stress [11]. The *Arabidopsis* RING-type E3, MIEL1, for example, has been shown to negatively modulate *Pseudomonas syringae* resistance by degrading MYB30, a positive regulator of hypersensitive cell death [16]. The overexpression of a RING-type E3 ligase gene *OsBBI1* in rice elevates broad-spectrum resistance to the blast fungus *Magnaporthe oryzae* by modifying cell wall defense responses [17]. RING-type E3 ligases also influence ABA-mediated stress (such as drought and salt) responses by participating in the degradation of positive or negative regulators in ABA biosynthesis and signaling [18,19,20]. However, the role of RING-type E3 ligase in plant herbivore resistance and its underlying mechanisms remain largely unknown.

Jumonji C (JmjC) domain-containing proteins, which have been identified to be ubiquitous histone demethylases in plants, can be divided into eight subfamilies: KDM6/JMJD3, KDM5/JARID1, KDM4/JHDM3, KDM3/JHDM2, KDM2/JHDM1, PHF, JMJD6, and JmjC domain-only. The JmjC domain is known to catalyze histone lysine demethylation via an oxidative reaction that needs Fe (II) and α-ketoglutarate (α-KG) as cofactors [21,22]. The occurrence of mutations in highly conserved cofactor-binding residues had led some to speculate that several JmjC proteins in *Arabidopsis* and rice are inactive histone demethylases [21,23,24]. Among them, JMJ24, a member of the *Arabidopsis* KDM3/JHDM2 subfamily, has been found unable to demethylate lysine 9 of histone H3 [24]. Interestingly, JMJ24 also contains two RING domains, and recent work by Deng et al. [25] has reported that JMJ24 directly interacts with and ubiquitinates a DNA methyltransferase, CHROMOMETHYLASE 3, indicating that JMJ24 has E3 ligase activity.

Rice, one of the most important grain crops in the world, suffers from many insect pests, including the piercing-sucking herbivore, brown planthopper (BPH, *Nilaparvata lugens*). Previous studies have found that BPH-induced defense responses in rice are regulated by a complex signaling network, which mainly includes MAPK cascades and pathways mediated by JA, SA, ET, and H_2_O_2_ [26,27,28,29,30]. However, whether RING-type E3 ligases are involved in the herbivore-induced defense responses of rice remains unclear.

To answer this question, we cloned a gene in rice, *OsJMJ715*, which contains a JmjC domain and a RING domain, and is induced by gravid BPH female infestation (according to transcriptome data in Xu et al. [31]). By combining molecular biology, biochemistry, and bioassays, we found that OsJMJ715 specifically localized into the nucleus and had ubiquitination activity. Moreover, OsJMJ715 negatively regulated the resistance of rice to BPH at least in part via inhibiting BPH-elicited ABA biosynthesis and callose deposition. These findings suggest that OsJMJ715 plays a crucial role in BPH-induced rice defenses.

## 2. Results

### 2.1. Isolating and Characterizing OsJMJ715

**Figure 1:**
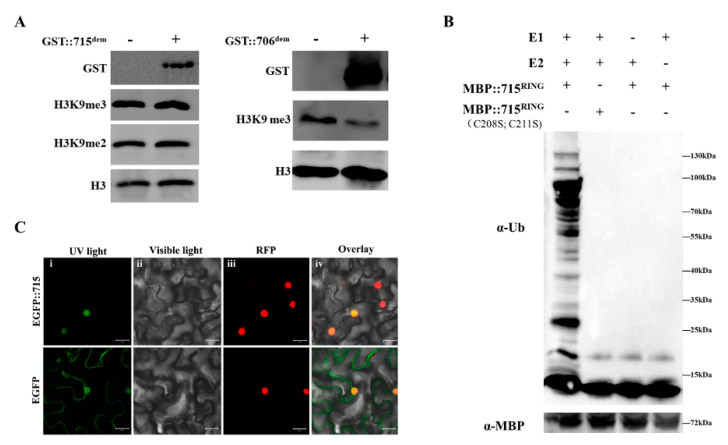
OsJMJ715 encodes a nucleus-localized RING-type E3 ubiquitin ligase. (**A**) In vitro histone demethylation assay of OsJMJ715. Bulk histone was incubated with (+) or without (-) GST::715^dem^ (left) or GST::706^dem^ (right) fusion proteins in the reaction buffer for 2 h at 30 °C and analyzed by immunoblotting by using anti-H3K9me2, anti-H3K9me3, anti-H3, and anti-GST antibodies. (**B**) In vitro ubiquitination assay of OsJMJ715. Fusion proteins of MBP::715^RING^ and its mutant form MBP::715^RING (C208S; C211S)^ were assayed for E3 activity in the presence of E1, E2, and ubiquitin (Ub). Anti-Ub and anti-MBP antibodies were used to detect ubiquitinated proteins and fusion proteins, respectively. Molecular weight markers (kDa) are shown on the right. (**C**) Subcellular localization of OsJMJ715. The EGFP::715 fusion protein and the EGFP protein were expressed in tobacco (*Nicotiana benthamiana*) leaves. Florescence assay was performed using a confocal microscopy. The photographs were taken in UV light, visible light, RFP, or in combination (overlay) (bar = 20 μm).

By using RT-PCR, the full-length cDNA of the *OsJMJ715*, including an open reading frame (ORF) of 3172 bp, was obtained (Figure S1). On the basis of the deduced amino acid sequence, we found that OsJMJ715 contains a JmjC domain at the C-terminal (Figure S2), belonging to the KDM3/JHDM2 group of JmjC protein family. Seeing that a lysine (K) in α-KG-binding sites was replaced by an alanine (A) (Figure S2), we realized that OsJMJ715 is probably an inactive histone demethylase, as predicted by Lu et al. [21]. To verify this hypothesis, we constructed the fusion protein containing glutathione S-transferase (GST) and jmjC domain of OsJMJ715 (GST::715^dem^) for an in vitro histone demethylation assay. Meanwhile, a jmjC domain of OsJMJ706 (a histone demethylase in rice) was used as a positive control. The results showed that in contrast to OsJMJ706, OsJMJ715 has no H3K9 demethylase activity (Figure 1A).

In addition to the JmjC domain, OsJMJ715 harbors a RING-finger domain at the N-terminal (Figure S2), which is known to function as ubiquitin E3 ligases [9]. To assess whether OsJMJ715 possesses E3 ligase activity, we expressed the RING domain of OsJMJ715 in *Escherichia coli* as a fusion with maltose-binding protein (MBP). In the presence of ubiquitin, E1 and E2, purified MBP::715^RING^ was able to self-ubiquitinate, whereas MBP fused with an RING mutant, 715^RING(C208S, C211S)^, which was constructed with substitutions of Cys-208 and Cys-211 with Ser-208 and Ser-211 in the RING domain, was unable to produce ubiquitinated bands (Figure 1B). These findings suggest that OsJMJ715 has E3 ligase activity, and the intact RING domain is required.

**Figure 2:**
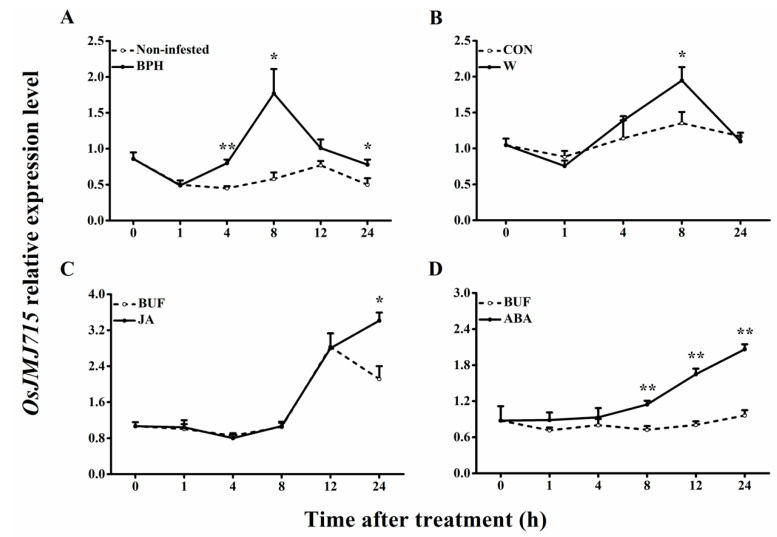
Expression profiles of *OsJMJ715* in rice leaf sheaths after different treatments. Mean transcript levels (+SE, *n* = 6) of *OsJMJ715* in rice leaf sheaths that were infested by brown planthopper (BPH) (**A**), mechanically wounded (W) (**B**), or treated with JA (**C**) or ABA (**D**). BUF: buffer; CON: non-manipulated controls. Asterisks indicate significant differences between treatments and controls at time points (* *p* < 0.05, ** *p* < 0.01, Student’s *t*-tests).

To elucidate the subcellular localization of OsJMJ715, we constructed an OsJMJ715::EGFP fusion gene, driven by a CaMV 35S promoter (Figure S3), and transiently expressed the construct in *Nicotiana benthamiana* leaves. The result showed that OsJMJ715 specifically localized to the nucleus (Figure 1C). We further performed quantitative real-time PCR to analyze the expression profiles of *OsJMJ715*. As shown in Figure 2, the transcript level of *OsJMJ715* increased in response to JA and ABA treatment (24 h and 8–24 h after treatment, respectively), mechanical wounding (8 h after treatment), and BPH infestation (4–24 h), especially at a late stage, after these treatments. The data suggest that OsJMJ715 may help regulate rice–herbivore interactions.

### 2.2. Silencing OsJMJ715

**Figure 3:**
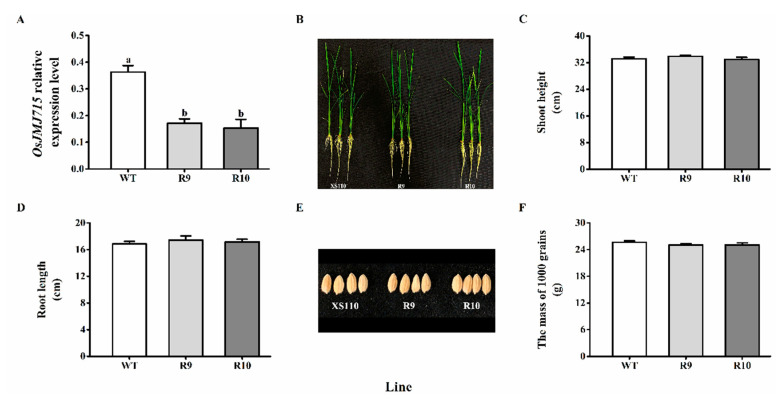
Silencing *OsJMJ715* did not influence rice growth. (**A**) Mean transcript levels (+SE, *n* = 6) of *OsJMJ715* in ir*JMJ* lines and wild-type (WT) plants that were mechanically wounded for 8 h. (**B**) Growth phenotype of 30-day-old ir*JMJ* and WT plants. (**C**,**D**) Mean shoot height (**C**) and root length (**D**) (+SE, *n* = 40) of 30-day-old ir*JMJ* and WT seedlings. (**E**,**F**) Growth phenotype (**E**) and mean weight (+SE, *n* = 6) of seeds (**F**) of ir*JMJ* and WT plants. Different letters indicate significant differences among ir*JMJ* and WT plants (*p* < 0.05, Tukey’s post hoc test).

To study the function of OsJMJ715 in the herbivore-induced rice defenses, we constructed *OsJMJ715*-RNAi transgenic plants, and obtained two independent T_2_ homozygous ir*JMJ* lines (R9 and R10) containing a single insertion (Figure S4). Transcriptional analysis showed that wounding-induced mRNA levels of *OsJMJ715* in R9 and R10 lines were only 47.3% and 42.3% of those in WT plants (Figure 3A). No obvious differences in growth phenotypes were observed between WT and transgenic lines (Figure 3B). Moreover, the shoot height, root length, seed size, and mass of R9 and R10 plants were all similar to those of WT plants (Figure 3C–F).

### 2.3. Silencing OsJMJ715 Enhanced the Resistance of Rice to BPH

**Figure 4:**
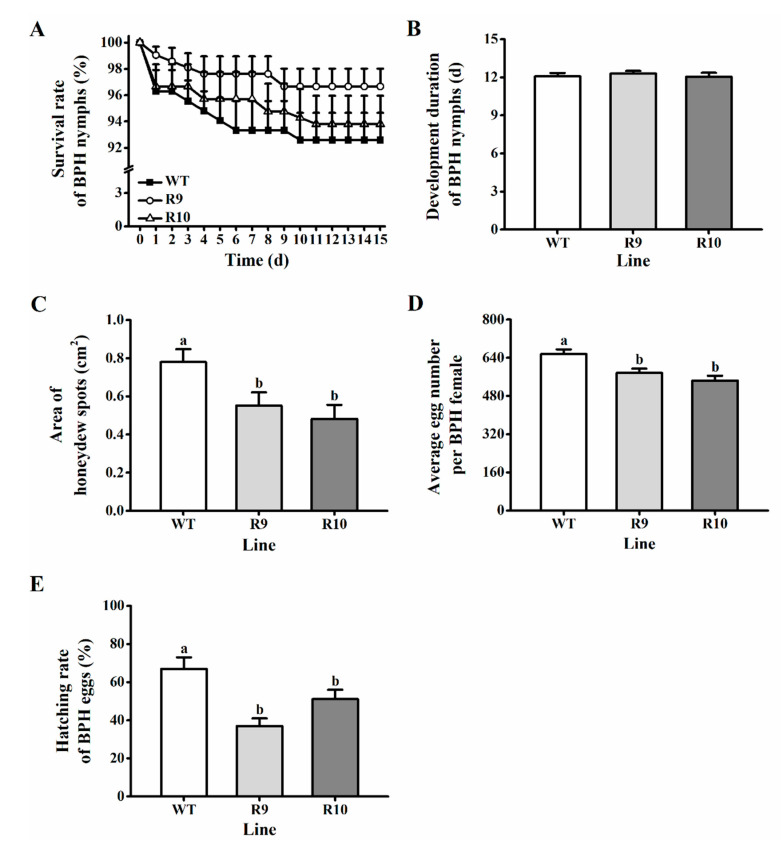
OsJMJ715 regulated rice resistance to brown planthopper (BPH). (**A**) Mean survival rate (+SE, *n* = 15) of 15 newly hatched BPH nymphs that fed on ir*JMJ* or wild-type (WT) plants within 15 days. (**B**) Mean developmental duration (+SE, *n* = 15) of BPH nymphs that fed on ir*JMJ* or WT plants. (**C**) Mean area of honeydew per day (+SE, *n* = 10) excreted by a BPH female adult that had emerged from ir*JMJ* or WT plants. (**D**) Mean number of eggs (+SE, *n* = 20) laid by a BPH female adult that had emerged from ir*JMJ* or WT plants. (**E**) Mean hatching rate (+SE, *n* = 15) of BPH eggs on ir*JMJ* or WT plants. Different letters indicate significant differences among ir*JMJ* and WT plants (*p* < 0.05, Tukey’s post hoc test).

To evaluate the effect of silencing *OsJMJ715* on the resistance of rice to BPH, we performed a series of bioassays. BPH nymphs fed on ir*JMJ* lines displayed similar survival rates and developmental durations to those fed on WT plants (Figure 4A,B). However, the amount of honeydew excreted by BPH female adults fed on R9 and R10 lines was significantly decreased, by 29.3% and 38%, respectively, compared to those that fed on WT plants (Figure 4C). Moreover, the number of eggs laid by female adults that had emerged from ir*JMJ* lines was less than that laid by female adults emerged from WT plants (Figure 4D). Silencing *OsJMJ715* also reduced the hatching rate of BPH eggs: for eggs laid on R9 and R10, the hatching rate was only 55.2% and 66.1%, respectively, of those laid on WT plants (Figure 4E). These results suggest that silencing *OsJMJ715* enhances the resistance of rice to BPH.

### 2.4. Silecing OsJMJ715 Enhanced BPH-Induced Levels of JA, JA-Isoleucine, and ABA

**Figure 5:**
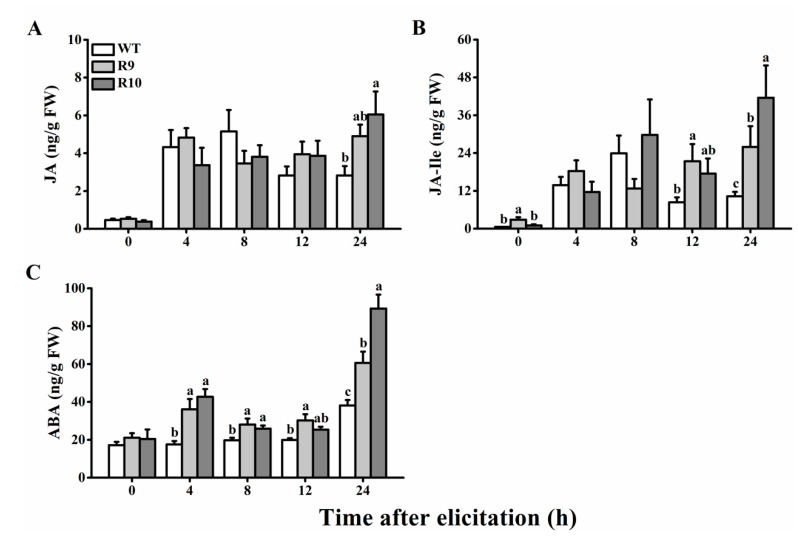
OsJMJ715 negatively mediated the brown planthopper (BPH)-induced accumulation of jasmonic acid (JA), JA-isoleucine (JA-Ile), and abscisic acid (ABA). Mean levels (+SE, *n* = 6) of JA (**A**), JA-Ile (**B**), and ABA (**C**) in ir*JMJ* lines and wild-type (WT) plants that were infested by 10 gravid BPH females. Different letters indicate significant differences among ir*JMJ* lines and wild-type (WT) plants at time points (*p* < 0.05, Tukey’s post hoc test).

To gain further insight into the mechanism underlying OsJMJ715-mediated rice resistance to BPH, we measured levels of JA, JA-isoleucine (JA-Ile), and ABA in WT and ir*JMJ* plants in response to gravid BPH female attack. As previously found [32], levels of JA and JA-Ile were induced quickly and continuously during gravid BPH female infestation (Figure 5A,B). Silencing *OsJMJ715* did not influence the constitutive level of JA but slightly enhanced the constitutive level of JA-Ile (Figure 5A,B). Infestations by BPH caused the level of both JA and JA-Ile in ir*JMJ* lines to rise, becoming significantly higher than that in WT plant 24 and 12–24 h, respectively, after its onset (Figure 5A,B). Gravid BPH female infestation also enhanced levels of ABA in both WT and ir*JMJ* plants (Figure 5C). Silencing *OsJMJ715* had no effect on constitutive levels of ABA, but it strengthened and quickened the response of ABA to BPH infestation—compared to that in non-infested plants, the level of ABA in BPH-infested plants began to significantly increase at 4 h after BPH attack in ir*JMJ* lines at 24 h in WT plants; moreover, the BPH-infested level of ABA in ir*JMJ* plants was always significantly higher than that in WT plants at 4–24 h after BPH infestation (Figure 5C). The data demonstrate that in plants silencing *OsJMJ715* enhances the response of JA, JA-Ile, and ABA to BPH infestation.

### 2.5. OsJMJ715 Negatively Mediated BPH-Elicited Callose Deposition

**Figure 6:**
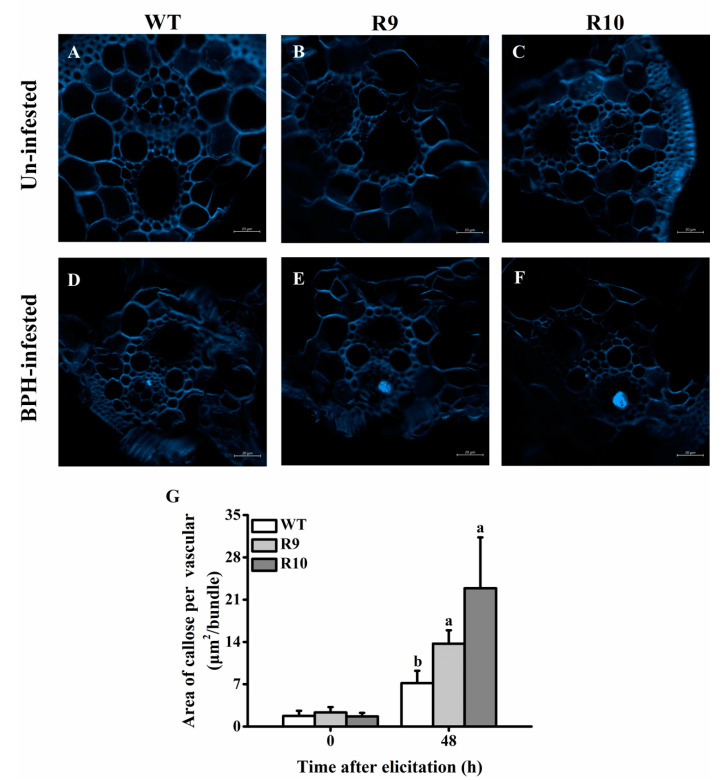
OsJMJ715 negatively mediated brown planthopper (BPH)-induced callose deposition. (**A**–**F**) Callose deposition in the vascular bundle in ir*JMJ* lines and wild-type (WT) plants without (**A**–**C**) or with infestation by 10 gravid BPH females for 48 h (**D**–**F**). Bright blue fluorescence indicates callose deposition (bar = 20 μm). (**G**) Mean area (+SE, *n* = 12) of callose per vascular bundle in ir*JMJ* lines or WT plants that were infested by one gravid BPH females for 48 h. Different letters indicate significant differences among ir*JMJ* lines and WT plants at time points (*p* < 0.05, Tukey’s post- hoc test).

Callose deposition in sieve plates is an important defense mechanism of rice plants to prevent BPH feeding [33]. To ask whether OsJMJ715 regulates callose deposition and thereby influences BPH feeding as stated above (Figure 4C), we investigated callose deposition in rice plants of different genotypes before and after BPH infestation. As previously reported [34,35], little callose was observed in non-infested plants (Figure 6A–C). Upon BPH attack for 48 h, callose deposition had increased in both WT and ir*JMJ* plants (Figure 6D–F); however, silencing *OsJMJ715* enhanced the deposition of callose—the amount of callose deposition in R9 and R10 was 1.9- and 3.2-fold, respectively, of that in WT plants (Figure 6G).

## 3. Discussion

The RING-type E3 ubiquitin ligases play an important role in plant growth and development as well as in defense responses to abiotic stresses and pathogens. In this study, we found that a rice RING-type E3 ubiquitin ligase, OsJMJ715, plays an important role in regulating the resistance of rice to BPH. Several lines of evidence support this statement. First, *OsJMJ715* was induced by BPH infestation and mechanical wounding. Second, silencing *OsJMJ715* enhances BPH-elicited accumulation of JA, JA-Ile, ABA, and callose, which subsequently reduces the hatching rate of BPH eggs and BPH feeding. Third, OsJMJ715 specifically localizes to the nucleus and has E3 ligase activity, suggesting that OsJMJ715 might be implicated in the degradation of proteins [36]. These data demonstrate that RING-type E3 ubiquitin ligase-mediated proteolysis is involved in regulating the resistance of plants to both pathogens [11,16,17,18,19,20] and herbivores (this study).

As stated in the introduction, RING-type E3 ubiquitin ligases have been reported to modulate phytohormone biosynthesis and signaling by degrading regulators in their processes. Here, we found that the expression of *OsJMJ715* was induced by BPH infestation, and treatment with JA and ABA, whereas silencing *OsJMJ715* enhanced BPH-induced levels of JA, JA-Ile, and ABA. This suggests that just as OsEBF1 attenuates *OsLOX9* expression by degrading its transcriptional activator OsEIL1, decreasing JA accumulation in rice [37], OsJMJ715 may degrade activators in the biosynthesis of JA, JA-Ile, and ABA when plants were infested by BPH; as such, OsJMJ715 may function as a negative regulator in the biosynthesis of BPH-induced JA, JA-Ile, and ABA by forming a negative feedback loop with the level of JA, JA-Ile, and ABA. Such a negative regulator, like OsNPR1 [38], 9-lipoxygenase (Osr9-LOX1) [39], OsWRKY53 [40], and MPK20-5 [41] reported previously in rice, may help ensure appropriate levels of JA, JA-Ile, and ABA in rice plants when infested by BPH, thereby avoiding the autotoxicity that excessive plant defenses may lead to. Further studies should identify the targets of OsJMJ715 under BPH infestation to uncover the mechanism underlying the OsJMJ715-mediated regulation of defensive phytohormone biosynthesis.

We observed that silencing *OsJMJ715* significantly reduced the amount of honeydew excreted by BPH and the hatching rate of BPH eggs. Given a significant increase in levels of BPH-induced ABA in ir*JMJ* plants, the above results suggest that ABA-mediated signaling pathway may play an important role in enhancing the resistance of rice to BPH. In addition to its contributions to plant disease resistance by controlling stomatal closure and callose deposition [42,43], the ABA-mediated signaling pathway has also been reported to regulate the resistance of plants to piercing-sucking herbivores, including BPH, by influencing the callose deposition on plant sieve plates, a mechanism by which plants effectively prevent piercing-sucking herbivores from ingesting phloem sap [33,44]. For instance, Zhou et al. [35] found that silencing the ABA hydrolase gene, *OsABA8ox3*, remarkably increased endogenous levels of ABA and callose deposition in rice, which in turn enhances the resistance of rice to BPH. Moreover, exogenous ABA increases the resistance of rice to BPH by promoting callose deposition, whereas resistant varieties that were treated with fluridone exhibit susceptibility to BPH with reduced callose deposition [45]. In addition to ABA-mediated signaling pathway, JA-mediated signaling pathway also plays an important role in modulating the resistance of rice to BPH by regulating the production of defensive compounds [26,31]. Thus, JA and ABA signaling pathways, both of which were strengthened in ir*JMJ* plants compared to WT plants, might contribute to the enhanced resistance of ir*JMJ* plants to BPH.

Some defensive compounds in rice against BPH have been reported. Callose deposition on sieve plates, for example, as stated above, inhibits BPH feeding [34,35,46]. Some flavonoids and phenolamides, such as sakuranetin, *p*-coumaroyl putrescine, feruloyl putrescine, and cinnamoyl putrescine, also affect the survival and development of BPH [47,48,49]. Although no ovicidal compounds for BPH eggs have been identified, benzyl benzoate in japonica rice has been demonstrated to have a lethal role on the eggs of the white-backed planthopper *Sogatella furcifera* [50]. Moreover, Zhou et al. [51] recently revealed that feeding on plants treated with benzyl benzoate decreases the fecundity of BPH female adults. We found that silencing *OsJMJ715* enhanced the accumulation of callose deposition. Hence, the decrease in BPH feeding on ir*JMJ* plants is at least in part related to the increase in callose deposition. We look forward to investigating whether other compounds are involved in influencing BPH feeding and which compounds are responsible for the decrease in the hatching rate of BPH eggs.

In summary, our results show that OsJMJ715, a RING-type E3 ligase, negatively regulates the biosynthesis of BPH-induced JA, JA-Ile, and ABA. BPH can benefit by inducing the expression of *OsJMJ715*, which subsequently decreases the level of BPH-induced ABA, JA, and JA-Ile, as well as the resulting defense responses in rice. These findings demonstrate that BPH reduces host plant resistance by regulating a RING-type E3 ligase.

## 4. Materials and Methods

### 4.1. Plant Material and Growth Conditions

The rice variety Xiushui 110 (XS110, japonica) and *OsJMJ715*-RNAi transgenic lines (using XS110 as the receptor variety) were used for this work. Water-soaked seeds were germinated in an incubator (28 ± 2 °C, 14 h light, 10 h dark). Seven-day-old seedings were transplanted to a hydroponic box (length × width × height: 45 × 30 × 15 cm) containing nutrient solution [52] and grown in a greenhouse (28 ± 2 °C, 14 h light, 10 h dark, 50–60% relative humidity) for 2 weeks, then were transplanted into individual plastic pots (diameter × height: 8 × 10 cm) that were placed in the greenhouse. Seven days later, the plants were used for experiments.

The transgenic tobacco (*N. benthamiana*) plants expressing the red fluorescent protein RFP-H2B were used for subcellular localization of OsJMJ715 [53]. Seeds were sown in a seedling pot (diameter × height: 5 × 8 cm) filled with a peat/vermiculite/perlite mixture (2:1:1, *v*/*v*/*v*) and grown in the greenhouse as mentioned above. Plants were watered weekly.

### 4.2. Insects

BPH colonies were originally obtained from rice fields in Hangzhou, Zhejiang province, China, and reared in a growth chamber (26 ± 2 °C, 14 h light) on seedlings of a rice variety TN1, which is susceptible to BPH.

### 4.3. Plant Treatments

For the mechanical wounding treatment, leaf sheaths of individual plant shoots were pierced 200 times with a needle (diameter 0.32 mm). Non-manipulated plants were used as controls. For BPH infestation, plants were individually confined in glass cylinders (diameter 4 cm, height 8 cm, with 48 small holes) into which 10 BPH gravid females were placed (Figure S5). Plants with empty cylinders were used as controls. For JA or ABA treatment, plants were grown in nutrient solution, and JA or ABA (Sigma-Aldrich, St. Louis, MO, USA) was added (first dissolved in a small volume of 70% (*v*/*v*) ethanol) into the nutrient solution to give a final concentration of 100 μM. Control plants were grown in nutrient solution without JA and ABA but with an equal volume of 70% ethanol. 

### 4.4. Isolation and Characterization of OsJMJ715

The full-length cDNA of *OsJMJ715* was obtained by reverse-transcription PCR from total RNA isolated from BPH-infested leaf sheaths of XS110 plants. The primers 715^ORF^-F1 and 715^ORF^-R1 (Table S1) were designed based on the sequence of *OsJMJ715* (accession number: Os03g31594). PCR-amplified fragments were cloned into the pEASY-Blunt Simple Vector (TransGen, Beijing, China) and sequenced (BioSune, Hangzhou, China).

Nucleotide sequence alignments were carried out using BLAST (https://blast.ncbi.nlm.nih.gov/Blast.cgi; accessed on 20 December 2015). Protein domains were analyzed using SMART (http://smart.embl-heidelberg.de/; accessed on 20 December 2015).

### 4.5. Quantitative Real-Time PCR

For RNA isolation, rice leaf sheaths were ground into fine powders in liquid nitrogen. Total RNA was isolated by the MiniBEST Plant RNA Extraction Kit (TaKaRa, Dalian, China). A total of 1 μg of RNA was reverse-transcribed using the PrimeScript^TM^ RT Master Mix (TaKaRa, Dalian, China). A quantitative real-time PCR assay was performed on a CFX96^TM^ Real-Time System (Bio-RAD, Hercules, CA, USA) using the TB Green^TM^ Premix EX Taq^TM^ II (Tli RNaseH Plus) (TaKaRa, Dalian, China). The rice actin gene *OsACT* (accession number: Os03g50885) was used as an internal standard to normalize cDNA concentrations in tested genes; primers used for qRT-PCR are provided in Table S2. Each treatment at each time point was replicated six times.

### 4.6. Subcellular Localization

The full-length open reading frame (ORF) of *OsJMJ715* without the stop codon was amplified by primers 715^ORF^-F2 and 715^ORF^-R2 (Table S1). The PCR product was inserted into p1301-EGFP vector [27] using a pair of restriction endonuclease, *Sal*I and *Bam*HI, to fuse it with EGFP (enhanced green fluorescent protein), yielding the construct OsJMJ715::EGFP (Figure S3). The construct was then transferred into *Agrobacterium tumefaciens* strain EHA105 competent cells, yielding a transformation vector. The vector was introduced into *N. benthamiana* leaves for transient expression as described in Huang et al. [53]. Tobacco leaves were cut into small pieces for fluorescence analysis at 24 h after agroinfiltration. The fluorescent signals were detected under a laser scanning confocal microscope (Zeiss, Oberkochen, Germany) as described by Huang et al. [53].

### 4.7. Generation of Transgenic Plants

A 382bp fragment of *OsJMJ715* was amplified by primers 715^RNAi^-F1/R1 and 715^RNAi^-F2/R2 (Table S1) and inserted into the pCAMBIA-1301 transformation vector to yield an *OsJMJ715*-RNAi interference construct (p1301-ir715, Figure S4). The construct was transferred into XS110 plants by using *A. tumefaciens*-mediated transformation. Homozygous lines of T_2_ generations were confirmed via GUS staining and PCR screening, as described in Zhou et al. [26]. Finally, two homozygous ir*JMJ* lines (R9 and R10) harboring a single insertion of T-DNA were confirmed by Southern blot analysis were selected for further studies.

### 4.8. Histone Demethylation Assay

The JmjC domains of *OsJMJ715* and *OsJMJ706* (accession number: Os03g31594) were amplified by primers 715^dem^-F/R and 706^dem^-F/R (Table S1), respectively [54]. Products were fused into the pGEX-4T-1 (GE Healthcare, Uppsala, Sweden) and then transferred into *E. coli* strain Transetta (DE3) (TransGen, Beijing, China). The recombinant proteins were purified by Glutathione Sepharose 4 Fast Flow (GE Healthcare, Uppsala, Sweden) according to the manufacturer’s instructions. An in vitro histone demethylation assay was performed as previously described by Whetstine et al. [55]. After the reaction, samples were analyzed by 10% SDS-PAGE followed by immunoblotting with anti-GST (GE Healthcare, Uppsala, Sweden), anti-H3, anti-H3K9me2, or anti-H3K9me3 antibodies (Abcam, Cambridge, UK).

### 4.9. Ubiquitination Assay

The RING domain of *OsJMJ715* was amplified by primers 715^RING^-F ansd 715^RING^ R (Table S1). The RING domain mutant, 715^RING(C208S^^, C211S)^, was constructed with substitutions of Cys-208 and Cys-211 with Ser-208 and Ser-211 in the RING domain using two rounds of overlapping PCR with primer pairs 715^RING^-F/715^C208S^-R, 715^C208S^-F/715^RING^-R (first round) and 715^RING^-F/715^C211S^-R, 715^C211S^-F/715^RING^-R (second round, Table S1). All of PCR products were cloned into pMAL-c5X (NEW ENGLAND BioLabs, Ipswich, MA, USA). Fusion plasmids were transferred into *E. coli* Transetta (DE3). The recombinant proteins were purified by Amylose Resin (New England, BioLabs) according to the manufacturer’s instructions. An in vitro ubiquitination assay was carried out as previously described in Xie et al. [56] and Zhang et al. [57]. A total of 20 ng recombinant proteins binding amylose resin beads, 50 ng E1, 400 ng E2, 5 μg ubiquitin, and 5 μg Mg^2+^-ATP were added to the reaction buffer containing 50 mM Tris (pH 7.4), 2 mM ATP, 5 mM MgCl_2_, and 2 mM DTT. The reaction mixture was incubated at 30 °C for 2 h at 900 rpm in a Thermomixer (Eppendorf, Hamburg, Germany). Reactions were stopped by adding loading buffer (Fdbio science, Hangzhou, China) and boiled for 8 min at 95 °C. Samples were analyzed by 8–16% SDS-PAGE (Genscript, Nanjing, China), followed by immunoblotting using anti-ubiquitin or anti-MYB (Abcam).

### 4.10. JA, JA-Ile, and ABA Measurement

Plants of different genotypes were randomly assigned to BPH and control treatments. For JA, JA-Ile and ABA analysis, outer leaf sheaths of plants were harvested at 0, 4, 8, 12 and 24 h after treatment. Samples (about 150 mg each) were ground in liquid nitrogen, compounds in each sample were extracted with 1 mL of ethyl acetate containing labeled internal standards (^2^D_2_-JA, ^2^D_6_-JA-Ile and ^2^D_6_-ABA) and analyzed by a Triple Quad liquid chromatography/mass spectrometry (Agilent Technologies, Santa Clara, USA) following the method described in Lu et al. [27]. Each treatment at each time point was replicated six times.

### 4.11. Callose Measurement

Rice plant shoots of different genotypes were individually confined within glass cylinders into which 10 gravid BPH females were placed. Then, 48 h later, outer leaf sheaths of the plants were collected, cut into 0.3–0.5 cm pieces, and immersed in 10% glycerol. Leaf sheaths were embedded in optimal cutting temperature compound (SAKURA, Torrance, CA, USA) and then cut into 10 μm thick slices via a microtome (Thermo, Waltham, MA, USA) and subsequently placed into 95% ethanol for overnight. Slices were soaked with 1/15 M phosphate buffer (pH 7.0) for 30 min, and then stained with 0.1% (*w*/*v*) aniline blue for 60 min. Samples were observed and photographed using a laser scanning confocal microscope (Zeiss) as described by Yang et al. [58]. Callose deposition area was analyzed by Image-Pro Plus (version 6.0). The callose deposition area of each bundle was calculated with 12 replicates for each treatment.

### 4.12. Herbivore Bioassays

To evaluate the effect of OsJMJ715 on the performance of BPH, we individually confined plants of wild-type (WT) and ir*JMJ* lines in cylinders into which 15 newly hatched BPH nymphs were released. The number of surviving nymphs and newly emerged adults on each plant were observed and recorded every day until all the nymphs had become adults. Each treatment was replicated 15 times. The newly emerged adults were then paired (one female with one male) and introduced into a new plant of the same genotype as they emerged. Fourteen days later, the number of BPH eggs in individual plants was counted under a microscope. The experiment was replicated 20 times. We also measured the amount of honeydew excreted by individual newly emerged BPH female adults using a method described in Paguia et al. [59]. Briefly, rice plant shoots were individually confined within transparent plastic cups (diameter 9 cm, height 12 cm) that were placed on plastic Petri dishes lined with filter paper (Figure S6). Individual newly emerged BPH female adults from different rice genotypes were released into the cups and allowed to feed for 24 h. The filter papers were collected and sprayed with a 0.1% ninhydrin in acetone solution [46] and then dried for 30 min at 60 °C. The area of the ninhydrin-positive deposition was measured with Image-Pro Plus. Each treatment was replicated 10 times. On the basis of these experiments, we calculated the survival rate and developmental duration of BPH nymphs, the fecundity of BPH female adults, and the amount of honeydew excreted by BPH female adults on plants of WT and ir*JMJ* lines.

To investigate the influence of OsJMJ715 on the hatching rate of BPH eggs, we individually infested plants of different genotypes by 10 gravid BPH female adults using the method above. Then, 24 h later, BPHs were removed. The number of newly hatched neonates was recorded every day until no neonates emerged for three days in a row. The number of unhatched eggs was counted under a microscope to calculate the hatching rate of BPH eggs. Each treatment was replicated 15 times.

### 4.13. Data Analysis

Differences between two comparisons were analyzed by Student’s *t*-tests. Multiple comparisons were analyzed by one-way ANOVAs followed by Tukey’s post hoc test. The normality of data was tested using the Kolmogorov–Smirnov test (*p* < 0.05), and the equality of error variances was tested by Levene’s test (*p* < 0.05). In the case of non-normality and/or unequal variances, data were logarithmic-transformed before ANOVAs. All data analysis was carried out with IBM SPSS Statistics, Version 20, International Business Machines Corporation (Armonk, NY, USA).

## Data Availability

Not applicable.

## Supplementary Materials

The following are available online at https://www.mdpi.com/article/10.3390/ijms222313020/s1.
