# Supplementary material for: Silencing an E3 Ubiquitin Ligase Gene OsJMJ715 Enhances the Resistance of Rice to a Piercing-Sucking Herbivore by Activating ABA and JA Signaling Pathways
Source: Int J Mol Sci. 2021 Dec 1;22(23):13020. doi: 10.3390/ijms222313020 (PMC8657654; doi:10.3390/ijms222313020)
Supplement: Supplementary file 1 [file ijms-22-13020-s001.zip › ijms-1462058-supplementary.pdf]

Supplementary Materials: Silencing an E3 Ubiquitin Ligase Gene *OsJMJ715* Enhances the Resistance of Rice to a Piercing-Sucking Herbivore by Activating ABA and JA Signaling Pathways

Yuebai Zhang, Mengting Chen, Shuxing Zhou, Yonggen Lou* and Jing Lu*

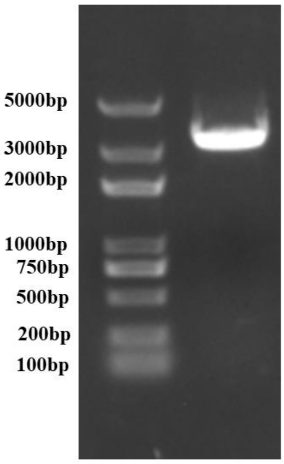

Figure S1. Gel electrophoresis of PCR amplified full-length of *OsJMJ715*.

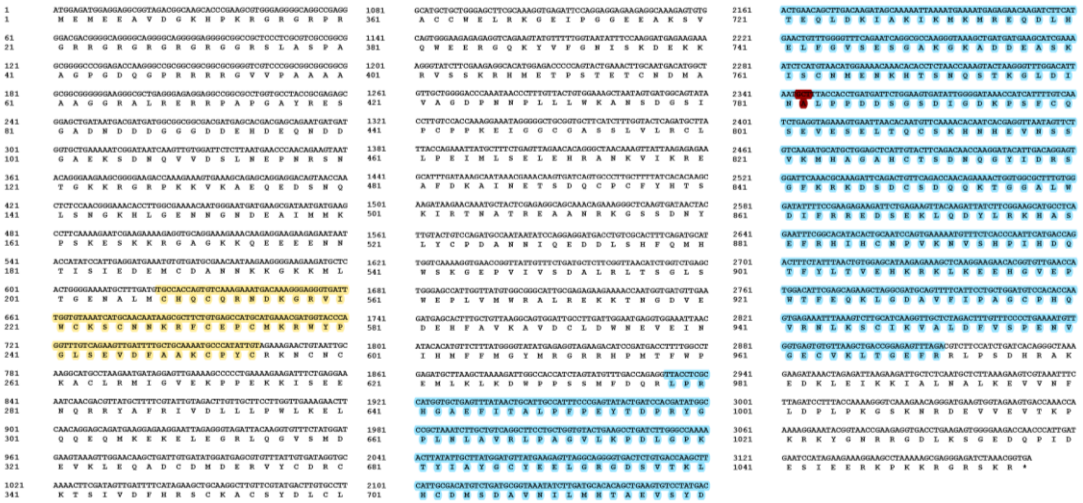

Figure S2. Sequences of nucleotides and deduced amino acids of *OsJMJ715*. The predicted RNIG domain is in yellow, JmjC domain is in blue, mutated site is noted in red, and the terminal codon is marked with an asterisk (*).

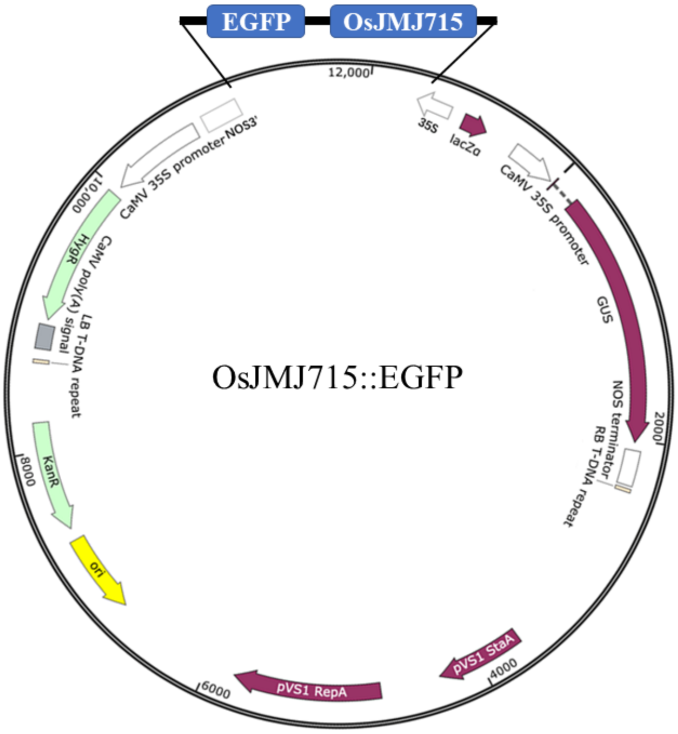

Figure S3. The OsJMJ715::EGFP vector used for subcellular localization.

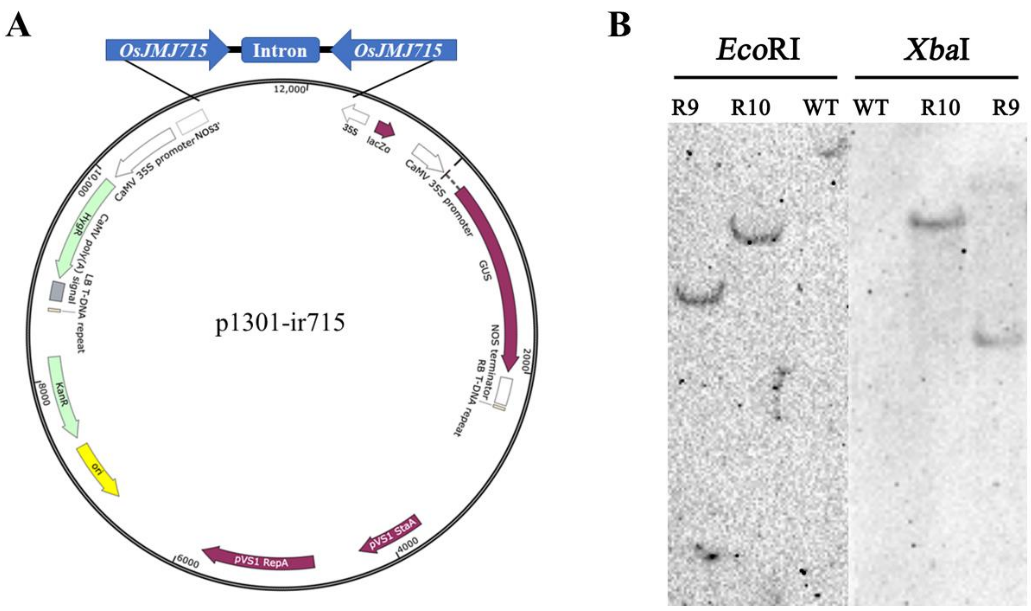

Figure S4. Generation of rice lines with silencing of *OsJMJ715*.
(**A**) The transformation plasmid used in this study. (**B**) DNA gel-blot analysis of ir*JMJ* lines and WT plants digesting with *Eco*RI or *Xba*I. The blot result was hybridized with a probe specific for *GUS* (a reporter gene).

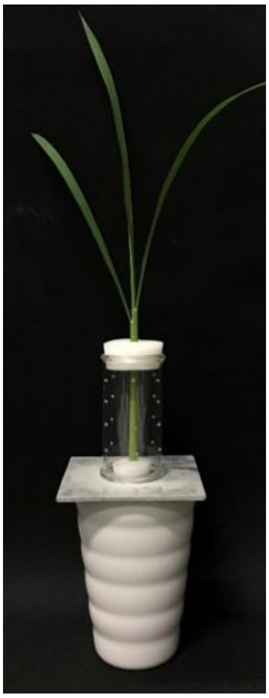

Figure S5. The experimental setup for BPH infestation.

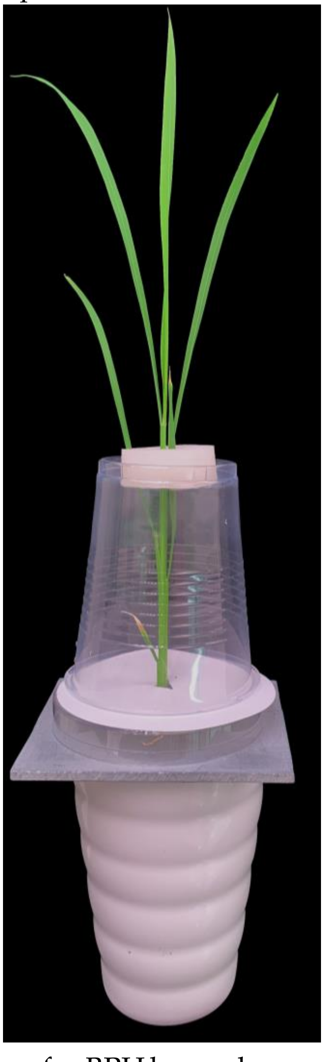

Figure S6. The experimental setup for BPH honeydew excretion measurement.

Table S1. Primers used for PCR.

Name	Sequence (5′-…-3′)
715^{ORF}-F1	CGGGATCCATGGAGATGGAGGAGG
715^{ORF}-R1	CGGAATTCTCACCGTTTAGATCTCCCT
715^{ORF}-F2	GGGGTCGACATGGAGATGG
715^{ORF}-R2	AAAGGATCCCCGTTTAGATCTCCCT
715^{RNAi}-F1	CTCTAGTTTATCAAGCTTATCGATGATGAGAAGAAAAGGGTACC
715^{RNAi}-R1	ACCGGGCCCCCCCTCGAGGTCGACCATTTGTTCTTATCTTGC
715^{RNAi}-F2	TGCTCTAGACATTTGTTCTTATC
715^{RNAi}-R2	AAAGGATCCGATGAGAAGAAAAG
715^{dem}-F	TCCCCCGGGATGTGCCACCAGTGTCAAAGAA
715^{dem}-R	ATAAGAATGCGGCCGCACGTCTAAACTCTCCGGTC
706^{dem}-F	TCCCCCGGGATGCAACAGGTG
706^{dem}-R	ATAAGAATGCGGCCGCCTAGGTTTGCTC
715^{RING}-F	AAAGGATCCATGGATGAGCAC
715^{RING}-R	AAAGAATTCTCCTGGAATCTCA
715^{C208S}-F	CACCAGTGTCAAAGAAATGACAAAG
715^{C208S}-R	CTTTGTCATTTCTTTGACACTGGTGACT
715^{C211S}-F	CAAAGAAATGACAAAGGGAGGGTGA
715^{C211S}-R	CCTCCCTTTGTCATTTCTTTGAGA

Table S2. Primers used for qRT-PCR.

Gene	TIGR ID	Forward primers (5′-…-3′)	Reverse primers (5′-…-3′)
OsACT	Os03g50885	TGGACAGGTTATCACCATTGGT	CCGCAGCTTCCATTCCTATG
OsJMJ715	Os03g31594	AGCTTCGCAAAGGTGAGATT	GACCTCTCTCTTCCCACTGC
